# Teeth Swallowed

**Published:** 1853-10

**Authors:** 


### TEETH SWALLOWED.

In our last, we noticed the case of a gentlemen having swallowed three teeth attached to a gold plate. These in a few weeks were discharged, having passed through the entire alimentary canal, and showed no evidences of digestion. The gold was of good quality. The teeth were two front and a lateral incisor attached to a bicuspid on each side.

